# Oral 5-HT3 receptor antagonist for at-home management of acute FPIES episodes in adults: A real-world observational study

**DOI:** 10.1016/j.waojou.2026.101473

**Published:** 2026-09-22

**Authors:** Sho Watanabe, Ayako Sato, Hiroto Seki, Tsunehito Yauchi, Kiwako Yamamoto-Hanada, Tatsuki Fukuie, Yukihiro Ohya, Ichiro Nomura

**Affiliations:** a Department of Gastroenterology, Soka Municipal Hospital, Saitama, Japan; b Allergy Center, National Center for Child Health and Development, Tokyo, Japan; c Department of Gastroenterology, Tokyo Metropolitan Bokutoh Hospital, Tokyo, Japan; d Department of Environmental and Occupational Health, Nagoya City University Graduate School of Medicine, Aichi, Japan; e Division of General Allergy, Bantane Hospital, Fujita Health University, Aichi, Japan; f Division of Eosinophilic Gastrointestinal Disorders, National Research Institute for Child Health and Development, Tokyo, Japan

**Keywords:** Accidental food exposure, Adult FPIES, Food protein–induced enterocolitis syndrome, Granisetron, 5-HT3 receptor antagonist

## Abstract

**Background:**

Food protein–induced enterocolitis syndrome (FPIES) is increasingly recognized in adults. Because adults encounter diverse food environments, ingestion of trigger foods may occur despite dietary avoidance. However, the real-world occurrence of post-diagnosis FPIES episodes and strategies for at-home management remain poorly described. We aimed to characterize post-diagnosis FPIES episodes in adults and evaluate the real-world use of orally administered granisetron as a patient-initiated rescue treatment.

**Methods:**

We conducted a retrospective observational study of adults diagnosed with FPIES between April 2023 and March 2025. Post-diagnosis FPIES episodes were recorded using patient diaries and a secure web-based reporting system. Patients were instructed to take granisetron (1 mg) when gastrointestinal symptoms developed after ingestion of a known trigger food. The primary outcome was time from granisetron intake to patient-reported symptom resolution. Time-to-event analyses were performed using Kaplan–Meier methods.

**Results:**

Thirty-three FPIES episodes in 12 adults were treated with granisetron. Abdominal distention (84.8%), abdominal pain (75.8%), nausea (63.6%), and diarrhea (39.4%) were the most common symptoms. The median interval from symptom onset to granisetron intake was 30 min, and the median time from intake to symptom resolution was 50 min. Kaplan–Meier analysis showed symptom resolution rates of 45.5% at 30 min, 81.8% at 60 min, and 87.9% at 90 min. No emergency transport or intravenous treatment was required, and only mild constipation occurred in 2 episodes.

**Conclusion:**

Oral granisetron may be associated with rapid symptom improvement and represent a potential patient-initiated option for at-home management of acute FPIES episodes in adults.

## Introduction

Food protein–induced enterocolitis syndrome (FPIES) is a non–IgE-mediated food allergy characterized by delayed gastrointestinal symptoms.^1^ Although classically described in children, FPIES is increasingly recognized in adults.^2^, ^3^, ^4^ Adult FPIES has been reported predominantly in women, with onset commonly occurring in the third to fourth decades of life.^3^, ^4^, ^5^, ^6^ Trigger foods most frequently include shellfish and other seafood, followed by egg and other foods.^3^, ^4^, ^5^, ^6^ In contrast to pediatric FPIES, which often presents with repetitive vomiting, adult FPIES more commonly manifests with severe abdominal pain and abdominal distention, accompanied by more heterogeneous symptoms such as nausea, vomiting, and diarrhea.^3^^,^^4^^,^^7^^,^^8^

Acute adult FPIES episodes can be distressing and difficult to manage in real-world settings because symptoms develop unpredictably after food ingestion and often occur outside medical supervision.^9^ Episodes typically follow a reproducible temporal pattern, with delayed onset after ingestion and spontaneous resolution over several hours; however, their real-world clinical course and management after diagnosis have not been well characterized.

In adults, the diagnosis is generally established based on clinical criteria and may be confirmed by oral food challenge (OFC).^3^^,^^10^ When OFC results are positive, strict elimination of the trigger food is recommended.^11^ Nevertheless, because adults are exposed to diverse foods and eating environments, complete avoidance is difficult, and accidental ingestion may occur even after diagnosis.^8^^,^^12^ Despite this clinical relevance, the frequency, clinical course, and management of post-diagnosis accidental ingestion events in adult FPIES have not been systematically evaluated.

Serotonin 5-HT3 receptor antagonists, including ondansetron and granisetron, have been used as adjunctive treatments for acute pediatric FPIES episodes, although supporting evidence remains limited.^2^^,^^13^ In adults, parenteral ondansetron has been reported to shorten symptom duration during OFC.^11^ However, the real-world use of orally administered 5-HT3 receptor antagonists as patient-initiated rescue therapy outside medical settings has not been described.

Because some adult FPIES episodes may be severe enough to require urgent medical care,^7^ practical strategies for at-home symptom management are needed. Therefore, this study aimed to characterize post-diagnosis FPIES episodes in adults and to evaluate the real-world use of an orally administered 5-HT3 receptor antagonist as a patient-initiated rescue strategy.

## Methods

### Study design and patient population

We conducted a single-center retrospective observational study based on prospectively collected clinical data from adults diagnosed with FPIES at a single institution in Japan between April 2023 and March 2025. Adult FPIES was diagnosed according to the criteria proposed by González-Delgado et al.^3^ These criteria included gastrointestinal symptoms alone (vomiting, acute abdominal pain, and/or diarrhea) occurring within 1–6 h after ingestion of the suspected trigger food, resolution with avoidance, recurrence on at least 2 occasions after the same or related foods, and previous tolerance to the trigger food. Food-specific IgE testing and/or skin prick testing were negative when clinically indicated. Other disorders, including food poisoning and anisakiasis, were excluded by gastroenterologic evaluation using clinical assessment, computed tomography, and endoscopy as appropriate.

Inclusion criteria were adult patients (aged ≥18 years) who visited our hospital during the study period and fulfilled the diagnostic criteria for adult FPIES. Exclusion criteria included patients with severe comorbidities such as advanced malignancies, or those who were unable to express their will, for whom the attending physician judged participation in the study inappropriate. No patients met the exclusion criteria during the study period.

### Oral food challenge and post-challenge management

Of the 41 patients in this study, OFC was performed on 31 patients. OFC was not performed on the remaining 10 patients because they had either recently experienced an FPIES event or were unwilling to consume the trigger food. The open-label OFC was conducted following the protocol, where the total serving size was divided into 2 portions. The first portion was 1/3 of the total portion, and the remaining portion was administered 1 h later, followed by a 4-h observation period. Because OFCs were performed under close medical supervision, oral granisetron jelly was not used. Instead, intravenous ondansetron was used as rescue therapy for all patients with positive OFC results. A positive OFC required fulfillment of all major criteria and at least 1 minor criterion.^11^ Major criteria included (1) ≥2 gastrointestinal symptoms (nausea, vomiting, abdominal pain, or abdominal distention) within 1–6 h after ingestion or severe abdominal pain alone (numerical rating scale ≥8), and (2) absence of skin or respiratory symptoms. Minor criteria included lethargy, marked pallor, hypotension, abnormal body temperature, cold sweats, diarrhea within 24 h, and neutrophilia (>1500/μL increase).

Because false-negative OFC results have been reported in FPIES,^14^ patients with negative OFC results were advised to reintroduce the food at home using the same amount and preparation as the OFC. Natural resolution was confirmed only after 3 consecutive uneventful home ingestions. In contrast, patients with positive OFC results and those who did not undergo OFC were advised to continue strict avoidance of the suspected trigger food.

### Definition of accidental FPIES events and granisetron use

Accidental FPIES episodes were defined as unintentional exposure to a previously identified trigger food followed by symptoms consistent with acute FPIES. No reactions to newly identified trigger foods were included in the present analyses. Episodes occurring during post-OFC home reintroduction were also recorded when symptoms recurred after a negative OFC.

Patients were instructed to take granisetron jelly (1 mg per sachet) as soon as gastrointestinal symptoms consistent with an acute FPIES reaction developed after ingestion of a known trigger food. A second dose was permitted if symptoms persisted.

### Data collection and statistical analysis

Accidental FPIES episodes were monitored after diagnosis from April 2023 through October 2025. In patients with negative OFC results, granisetron use during symptom-provoking episodes occurring at home after food reintroduction was also recorded and included in the analyses. Event-level data were recorded in real time using structured patient diaries and a secure web-based reporting system as part of routine clinical care. These routinely collected clinical data were retrospectively analyzed in the present study. Collected variables included the suspected trigger food, gastrointestinal symptoms, timing of symptom onset, timing of granisetron intake, time to complete symptom resolution, need for emergency medical care or intravenous treatment, repeat dosing, and adverse events. The primary outcome was the time from granisetron intake to patient-reported symptom resolution, defined as the complete disappearance of gastrointestinal symptoms. Patients were instructed to continue monitoring for at least 24 h to capture delayed or recurrent symptoms. Adverse events were graded according to the Common Terminology Criteria for Adverse Events (CTCAE), version 5.0.

Time to symptom resolution after granisetron intake was analyzed using Kaplan–Meier methods with 95% confidence intervals. Symptom-specific improvement rates were calculated at 30, 60, and 90 min using the Wilson method. Exploratory within-patient comparisons of episode duration with and without granisetron were performed using geometric mean ratios.

All statistical analyses were performed using GraphPad Prism (GraphPad Software Inc., San Diego, CA, USA) and R software (version 4.4.2) with custom R scripts. Given the exploratory design of the study and the possibility of multiple events within the same patient, analyses were primarily descriptive.

### Ethics

This study was approved by the Ethics Committee of Soka Municipal Hospital (approval nos. 165 and R7-23) and was conducted in accordance with the Declaration of Helsinki and Good Clinical Practice guidelines. Written informed consent was obtained from all participants.

## Results

### Study population and event ascertainment

The study flow and event ascertainment process are shown in Fig. 1. Among 41 adults diagnosed with FPIES, 31 underwent OFC: 15 (48.4%) were positive, 14 (45.2%) negative, and 2 (6.5%) indeterminate. Among patients with negative OFC results, 10 of 14 (71.4%) subsequently confirmed resolution after repeated home intake, whereas 4 of 14 (28.6%) developed symptoms during post-OFC home reintroduction and were classified as having active FPIES. Among 17 patients with positive or indeterminate OFC results who continued dietary elimination, 9 (52.9%) experienced accidental FPIES events and 8 (47.1%) remained event-free.

**Fig. 1:**
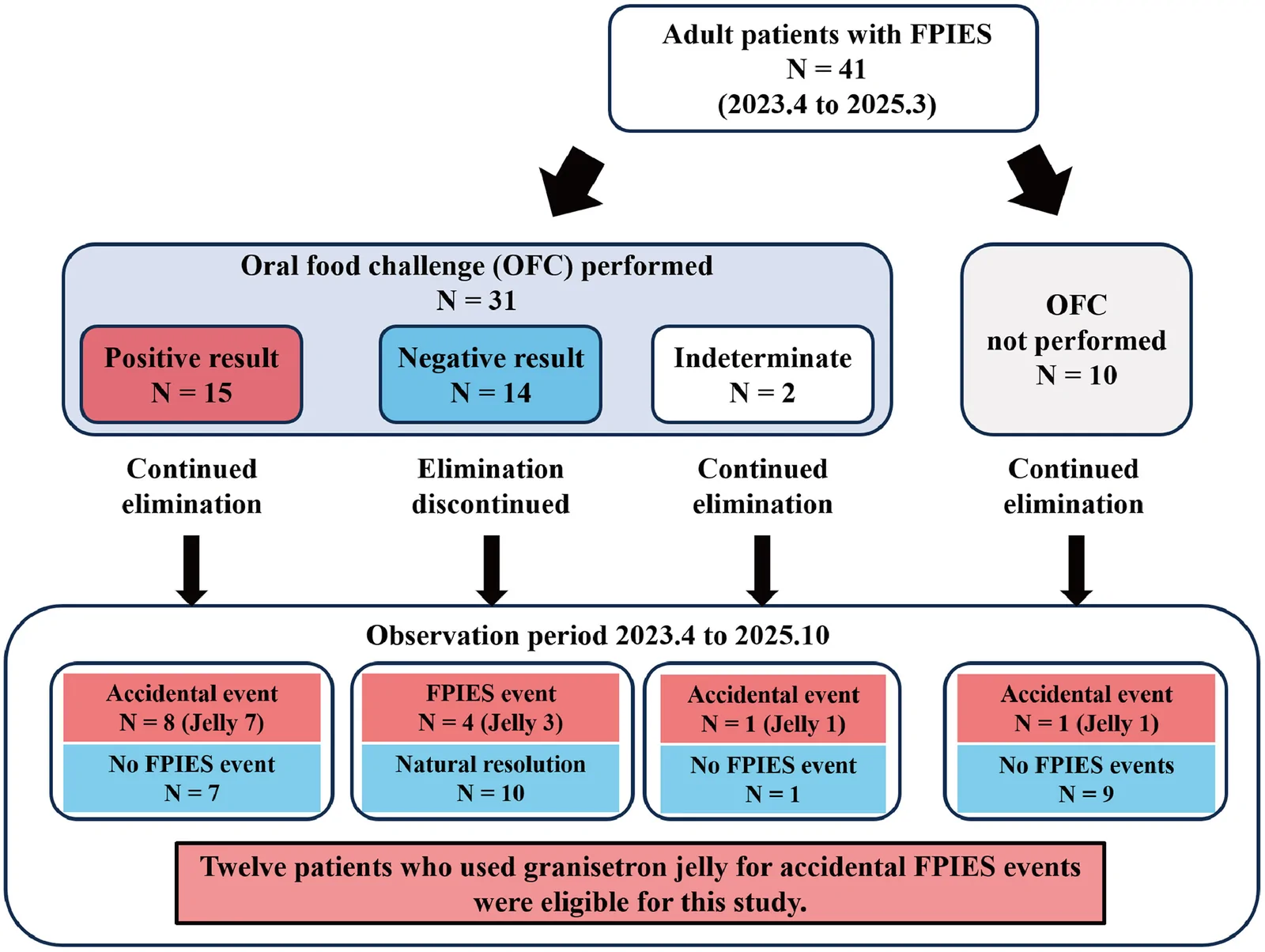
**Elimination strategies and outcomes in adult FPIES according to OFC status**. Adult patients with FPIES (N = 41) were categorized based on whether OFC was performed. Continued or discontinued elimination and subsequent accidental FPIES events, granisetron jelly use, and natural resolution are shown. The number in parentheses labeled “Jelly” indicates the number of individual patients who used oral granisetron jelly for accidental FPIES episodes

Two patients with very mild symptoms did not use granisetron jelly and were excluded from granisetron-related analyses. Among 10 patients who did not undergo OFC, 1 experienced an accidental FPIES episode treated with granisetron, whereas 9 had no subsequent episodes.

Overall, 33 FPIES episodes in 12 patients—including accidental ingestion of known trigger foods and symptom-provoking episodes during post-OFC home reintroduction—were treated with granisetron jelly (Fig. 1).

### Patient characteristics

Patient characteristics are summarized in Table 1. The median age at FPIES onset was 27.5 years (IQR, 20.5–33.5), and the median age at study enrollment was 39.5 years (IQR, 31.0–43.0). Most patients were female (83.3%). Allergic rhinitis was the most common atopic comorbidity, affecting 10 patients (83.3%), whereas IgE-mediated food allergy was present in 3 patients (25.0%). Functional gastrointestinal disorders were reported in 4 patients (33.3%). Seafood was the most common offending food category, reported in 7 patients (58.3%), followed by vegetables in 6 patients (50.0%). Before identification and elimination of trigger foods, patients experienced a median of 50 FPIES episodes (IQR, 10.0–100.0). The median duration of typical untreated FPIES episodes (without granisetron use) was 13.0 h (IQR, 8.3–24.0).

**Table 1:** Patient characteristics of adults with FPIES who used granisetron (n = 12.)

Characteristic (total 12 patients)	Summary (n [%] or median [IQR])
Age (years), median [IQR]	39.5 [31.0–43.0]
Sex (Male/Female), n (%)	2 (16.7%)/10 (83.3%)
Body mass index, median [IQR]	21.7 [18.7–22.1]
Age at onset of FPIES (years), median [IQR]	27.5 [20.5–33.5]
Atopic comorbidity (Rhinitis/Dermatitis/Asthma), n (%)	10 (83.3%)/5 (41.7%)/3 (25.0%)
Other food allergy (IgE-mediated), n (%)	3 (25.0%)
Offending food category, n (%)	Seafood 7 (58.3%); Vegetables 6 (50.0%); Egg 2 (16.7%); Fruit 2 (16.7%); Meat 2 (16.7%); Grains 2 (16.7%); Milk 1 (8.3%); Nuts 1 (8.3%)
Specific IgE positive/skin prick test positive, n (%)	0 (0%)/0 (0%)
Reacting to >1 food group, n (%)	5 (41.7%)
Reacting to >1 food from the same food group, n (%)	6 (50.0%)
Past medical history, n (%)	Functional gastrointestinal disorders 4 (33.3%); Psychiatric disorders 1 (8.3%); Migraine 1 (8.3%); Graves' disease 1 (8.3%); Renal disease on hemodialysis 1 (8.3%); Gastroesophageal reflux disease 1 (8.3%); Other 1 (8.3%)
Number of acute episodes experienced, median [IQR]	50.0 [10.0–100.0]
Attack duration (hours), median [IQR]	13.0 [8.3–24.0]

Values are expressed as n (%) or median [IQR]. IQR, interquartile range. Other comorbidities included ovarian cysts, appendicitis, gallstones, gallbladder polyps, intraductal papillary mucinous neoplasm, and retinal detachment

### Characteristics of granisetron-treated episodes

Characteristics of the 33 granisetron-treated episodes are summarized in Table 2. Detailed de-identified episode-level data are provided in Supplemental Table 1. Seafood was the most common offending food category, accounting for 10 episodes (30.3%), followed by vegetables (8, 24.2%), grains (5, 15.2%), and egg (4, 12.1%). Abdominal distention (28 episodes, 84.8%), abdominal pain (25, 75.8%), nausea (21, 63.6%), and diarrhea (13, 39.4%) were the most common symptoms, whereas vomiting was observed in 4 episodes (12.1%). Notably, 22 events (66.7%) occurred at night and 26 events (78.8%) occurred on weekdays. No emergency transport or intravenous infusion was required, and mild constipation (CTCAE grade 1) was reported in 2 episodes (6.1%).

**Table 2:** Characteristics of accidental FPIES episodes treated with granisetron (33 events in 12 patients).

FPIES event's characteristics (total 33 events)	Summary (n [%] or median [IQR])
Age (years), median [IQR]	39.5 [31.0–43.0]
Sex (Male/Female), n (%)	2 (16.7%)/10 (83.3%)
Offending food category, n (%)	
Seafood	10 (30.3%)
Vegetable	8 (24.2%)
Grain	5 (15.2%)
Egg	4 (12.1%)
Meat	4 (12.1%)
Milk	1 (3.3%)
Soy	1 (3.3%)
Predominant symptoms at onset, n (%)	
Abdominal distention	28 (84.8%)
Abdominal pain	25 (75.8%)
Nausea	21 (63.6%)
Lethargy	18 (54.5%)
Diarrhea	13(39.4%)
Cold sweat	7 (21.2%)
Symptom onset → granisetron intake (min), median [IQR]	30 [7–60]
Symptom resolution after intake (min), median [IQR]	50 [20–60]
Total attack duration (min), median [IQR]	64 [50–107]
Time of day, n (%)	Day 11 (33.3%), Night 22 (66.7%)
Day type, n (%)	Weekday 26 (78.8%), Holiday 7 (21.2%)
Concomitant medication, n (%)	None (100%)
Adverse events, n (%)	Constipation (CTCAE G1) in 2 events (6.1%)
Emergency visit/IV infusion required, n (%)	0 (0%)

Values are expressed as n (%) or median [IQR]. Age and sex are summarized at the patient level for the 12 patients who contributed the 33 accidental FPIES episodes. All other variables are summarized at the event level. Seafood included clam, shrimp, crab, lobster, squid, sardine, fish (species not identified), herring roe, and pollock roe. Vegetables included garlic and onion. Grain included wheat. Daytime was defined as 06:00–17:59 and nighttime as 18:00–05:59. Weekdays were Monday–Friday, and holidays were Saturday, Sunday, and national holidays in Japan. IQR, interquartile range

### Time to symptom resolution after granisetron

Across granisetron-treated episodes, the median interval from symptom onset to granisetron intake was 30 min. The median time from granisetron intake to patient-reported complete symptom resolution was 50 min (Table 2). Kaplan–Meier analysis demonstrated cumulative complete symptom resolution rates of 45.5% at 30 min, 81.8% at 60 min, and 87.9% at 90 min after granisetron administration (Fig. 2). Most episodes resolved within 60 min of granisetron administration.

**Fig. 2:**
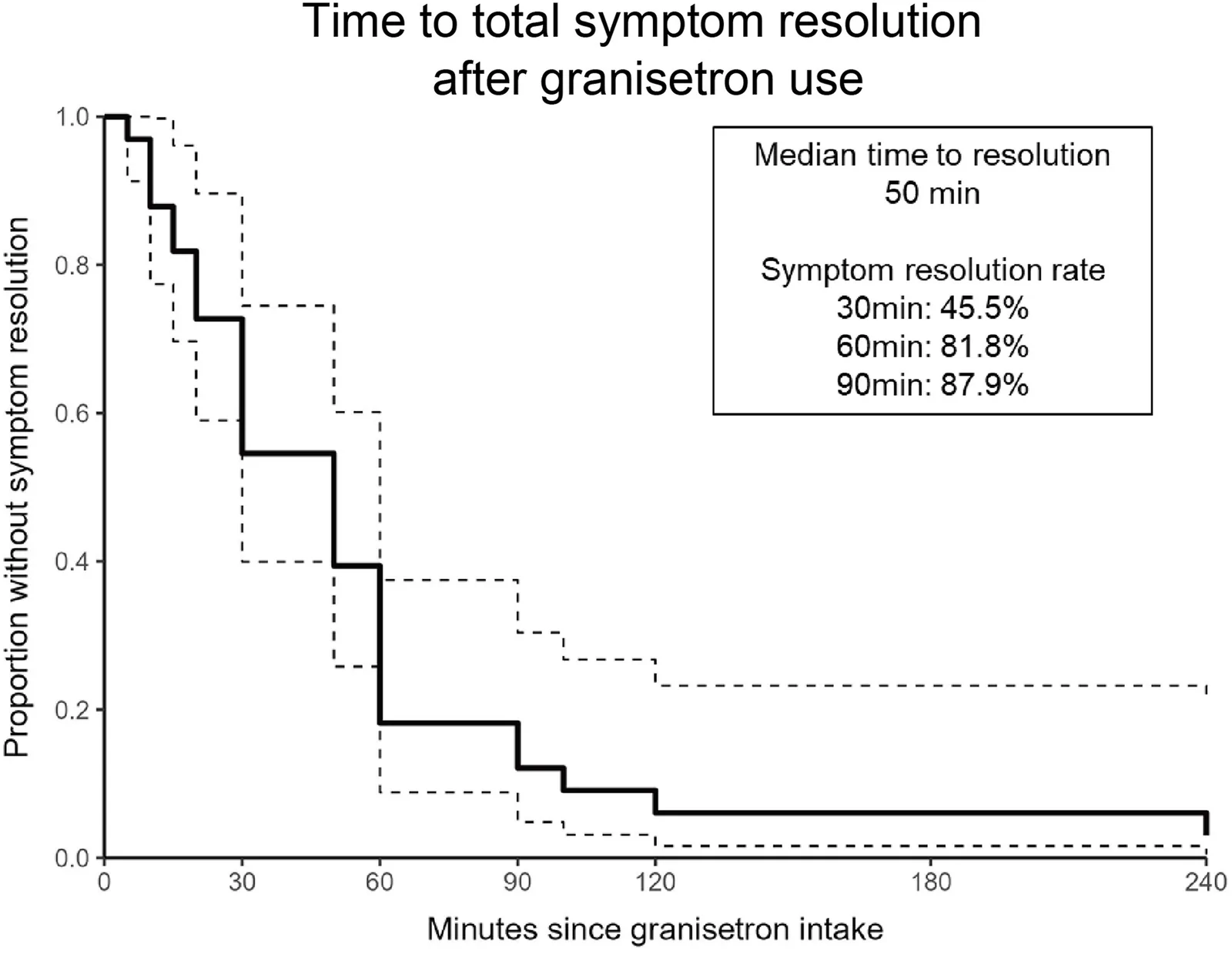
**Time course of symptom resolution after granisetron use during acute FPIES episodes**. Kaplan–Meier estimates show time from granisetron intake to patient-reported complete symptom resolution for 33 FPIES episodes (median, 50 min; dotted lines indicate 95% confidence intervals)

### Symptom-specific improvement

Symptom-specific improvement after granisetron use was observed across multiple gastrointestinal symptoms (Fig. 3). Nausea, abdominal pain, abdominal distention, and diarrhea improved rapidly, with approximately 70%–80% of episodes showing improvement within 60 min. Diarrhea showed the fastest early response, with improvement in about 70% of episodes within 30 min. Abdominal pain improved in more than half of episodes within 30 min. In contrast, vomiting improved more gradually, with 25% of episodes improving within 30 min and 50% within 60 min. Overall, most symptoms improved within 60 min after granisetron administration.

**Fig. 3:**
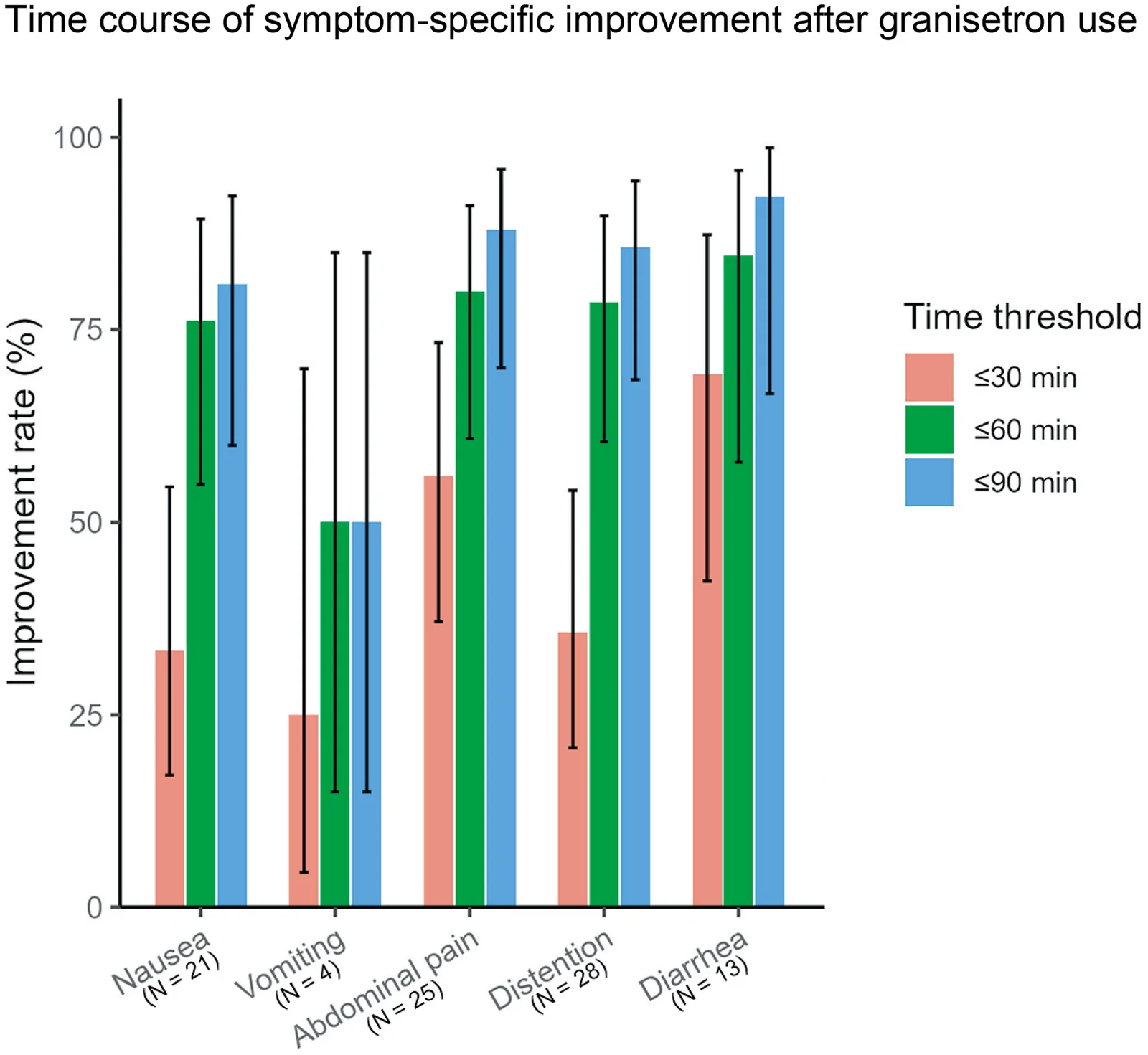
**Symptom-specific improvement after granisetron use during acute FPIES episodes**. Bar graphs show the proportions of events in which each abdominal symptom improved at 30, 60, and 90 min after granisetron use (33 events in 12 patients). Error bars represent 95% confidence intervals calculated using the Wilson method

### Exploratory within-patient comparison

In exploratory within-patient comparisons, granisetron-treated episodes appeared shorter than patients’ self-reported untreated episodes, although these findings should be interpreted cautiously because untreated episode duration was retrospectively reported (Supplemental Fig. 1).

## Discussion

This study provides the first detailed description of real-world use of an orally administered 5-HT3 receptor antagonist for self-management of acute FPIES episodes in adults. Granisetron use was followed by rapid symptom improvement, with most gastrointestinal symptoms resolving within approximately 60 min after intake. These findings suggest that an oral 5-HT3 receptor antagonist may represent a potential option for at-home management of acute FPIES episodes in adults.

Accidental exposure remained common even after diagnosis. The predominance of women in this cohort was consistent with previous reports describing a female predominance in adult FPIES.^15^, ^16^, ^17^ In this cohort, accidental ingestion occurred in 9 of 17 patients (52.9%) who continued dietary elimination after positive or indeterminate OFC, and symptom recurrence during post-OFC home reintroduction occurred in 4 of 14 patients (28.6%). These findings support the importance of confirming resolution by repeated home reintroduction rather than relying solely on a negative OFC, because false-negative OFC results have been reported in patients with FPIES.^14^ Notably, many episodes occurred during weekday evenings, when access to medical care may be limited. Previous epidemiological studies have shown that adults with FPIES frequently encounter delays in diagnosis and limited disease recognition in clinical practice.^9^^,^^18^ In addition, health-related quality-of-life studies have demonstrated that adult FPIES patients often experience anxiety related to the unpredictability and potential severity of acute attacks.^9^^,^^12^^,^^19^ Seafood was the most common offending food category, consistent with previous reports of adult FPIES,^18^^,^^20^ whereas vegetables were the second most frequent trigger in the present cohort. The relatively high frequency of accidental exposures observed in this cohort may reflect the practical challenges of complete dietary avoidance in adults, who frequently encounter restaurant meals, take-out foods, and processed foods containing hidden ingredients in daily life.^8^ These findings underscore the importance of establishing practical strategies for at-home management of FPIES episodes. The clinical manifestations observed during accidental home episodes were generally similar to those observed during supervised positive OFCs, although food exposure and rescue treatment differed between the 2 settings (Supplemental Table S2).

5-HT3 receptor antagonists have been reported to improve symptoms in pediatric FPIES.^13^^,^^21^ Pediatric FPIES often presents as a relatively homogeneous phenotype dominated by repetitive vomiting,^2^ whereas adult FPIES may involve more heterogeneous gastrointestinal manifestations, including abdominal pain, distention, vomiting, and diarrhea.^3^^,^^18^ In adults, parenteral ondansetron has been associated with shorter symptom duration during oral food challenges, with a median time to symptom resolution of approximately 1 h.^11^ In the present study, oral granisetron jelly was not used during OFCs because intravenous ondansetron served as the standard rescue treatment for positive OFC reactions under close medical supervision. Although a descriptive comparison of the clinical manifestations is provided (Supplemental Table S2), treatment outcomes were not directly compared because of differences in treatment setting, route of administration, and food exposure conditions. However, data regarding oral agents for self-administration outside medical settings have been lacking. The present study therefore extends previous findings by demonstrating the real-world applicability of an orally administered 5-HT3 receptor antagonist.

Imaging and translational studies may provide biological plausibility for the observed effects. In pediatric FPIES, abdominal ultrasonography has demonstrated small-intestinal dilation during acute episodes,^22^ while CT imaging studies in adults have similarly shown small-bowel dilation associated with abdominal symptoms.^11^ Furthermore, transcriptomic analyses of gastric and duodenal mucosa in adults with FPIES have suggested increased neuroendocrine signaling pathways in non-remission cases.^23^ Because serotonin signaling plays an important role in enteric neural pathways and intestinal motility,^2^^,^^21^ 5-HT3 receptor blockade may alleviate not only nausea and vomiting but also abdominal pain, distention, and diarrhea. According to the product information, after oral administration of 2 mg granisetron jelly in healthy adults, the time to maximum plasma concentration is approximately 2.1 h and the elimination half-life is approximately 4.1 h. Granisetron selectively binds to the 5-HT3 receptor with high affinity, thereby inhibiting serotonin-mediated reflex responses.^24^ Consistent with this hypothesis, exploratory within-patient comparisons suggested markedly shorter overall episode duration with granisetron than with retrospectively reported untreated episodes (Supplemental Fig. S1). Although the untreated episodes were assessed retrospectively, the substantial reduction in episode duration provides important supportive evidence for the effectiveness of oral granisetron in the absence of a prospective untreated control group.

The relatively slower improvement of vomiting observed in this study may have been influenced by several factors. Given that the time to maximum plasma concentration of oral granisetron jelly is approximately 2.1 h, the delayed improvement in vomiting may partly reflect its pharmacokinetic profile. In addition to reduced oral drug absorption when vomiting occurred before or shortly after administration, granisetron may have been administered later during the clinical course in some patients. Another possible explanation is that, in adults, episodes accompanied by vomiting may represent a relatively more severe clinical presentation than episodes characterized predominantly by abdominal symptoms. Because only a limited number of vomiting episodes were available for analysis, these hypotheses remain speculative and require confirmation in larger studies.

This study has several limitations. It was retrospective and single-arm, and outcomes relied on patient-reported symptom resolution because episodes occurred outside medical supervision. The sample size was modest, and the cohort may have preferentially included patients with frequent prior episodes, lower reaction thresholds, or lifestyles associated with higher exposure risk. The distribution of offending foods may also have been influenced by regional dietary habits and food environments, which may limit generalizability to other populations. Untreated episode duration was based on representative past events rather than prospectively collected data. The interval from symptom onset to granisetron intake may have influenced the observed time to symptom resolution, because earlier administration may have occurred in milder or more readily recognized episodes. Finally, multiple events per patient may have introduced within-subject correlation, which was not formally accounted for in the analyses.

Despite these limitations, this study provides the first real-world description of patient-reported outcomes with an oral, self-administered medication for acute FPIES episodes in adults. Adverse events were limited to mild constipation, supporting the potential feasibility of at-home use. However, despite these encouraging findings, additional evidence is needed before oral 5-HT3 receptor antagonists can be more widely recommended for routine management of acute FPIES episodes. Theoretical concerns have been raised regarding antiemetic use in acute gastrointestinal conditions, although supporting clinical evidence remains limited. This is consistent with previous evidence summaries evaluating ondansetron for acute gastroenteritis, which concluded that further evidence is required before routine clinical use can be recommended.^25^ Appropriate patient education will be essential, and oral 5-HT3 receptor antagonists may be best targeted to episodes occurring after suspected or confirmed ingestion of a known trigger food and presenting with symptoms consistent with the patient's typical FPIES phenotype. If confirmed in future studies, this approach may help reduce unnecessary emergency visits, intravenous treatment, and empiric systemic corticosteroid use for acute FPIES episodes. Larger prospective, controlled studies are warranted to further evaluate the effectiveness and safety of granisetron and to identify predictors of accidental exposure after diagnosis.

## Disclosure of the use of generative AI and AI-assisted technologies

The authors used ChatGPT (OpenAI) solely for language refinement. All scientific content, interpretation, and final decisions were made by the authors.

## Authors’ contributions

SW designed the study. SW, AS, HS, and IN collected and analyzed the data. SW wrote the manuscript. AS, TY, KY, TF, YO, and IN supervised and critically reviewed the manuscript. All authors have read and approved the final manuscript.

## Statement of ethics

This study was approved by the Ethics Committee of Soka Municipal Hospital (Approval no. 165 and R7-23) and was conducted in accordance with the Declaration of Helsinki, good clinical practice, and local regulations. Written informed consent was obtained from all participants.

## Disclosures

The authors declare no conflicts of interest.

## Funding

This work was supported by 10.13039/501100001691JSPS KAKENHI (Grant Number 25K19282) and the JSA-WAO Research Grant 2026 (to S. Watanabe).
