# Supplementary material for: Oral 5-HT3 receptor antagonist for at-home management of acute FPIES episodes in adults: A real-world observational study
Source: World Allergy Organ J. 2026 Sep 22;19(10):101473. doi: 10.1016/j.waojou.2026.101473 (PMC13651486; doi:10.1016/j.waojou.2026.101473)
Supplement: Supplementary file 1 [file mmc1.pdf]

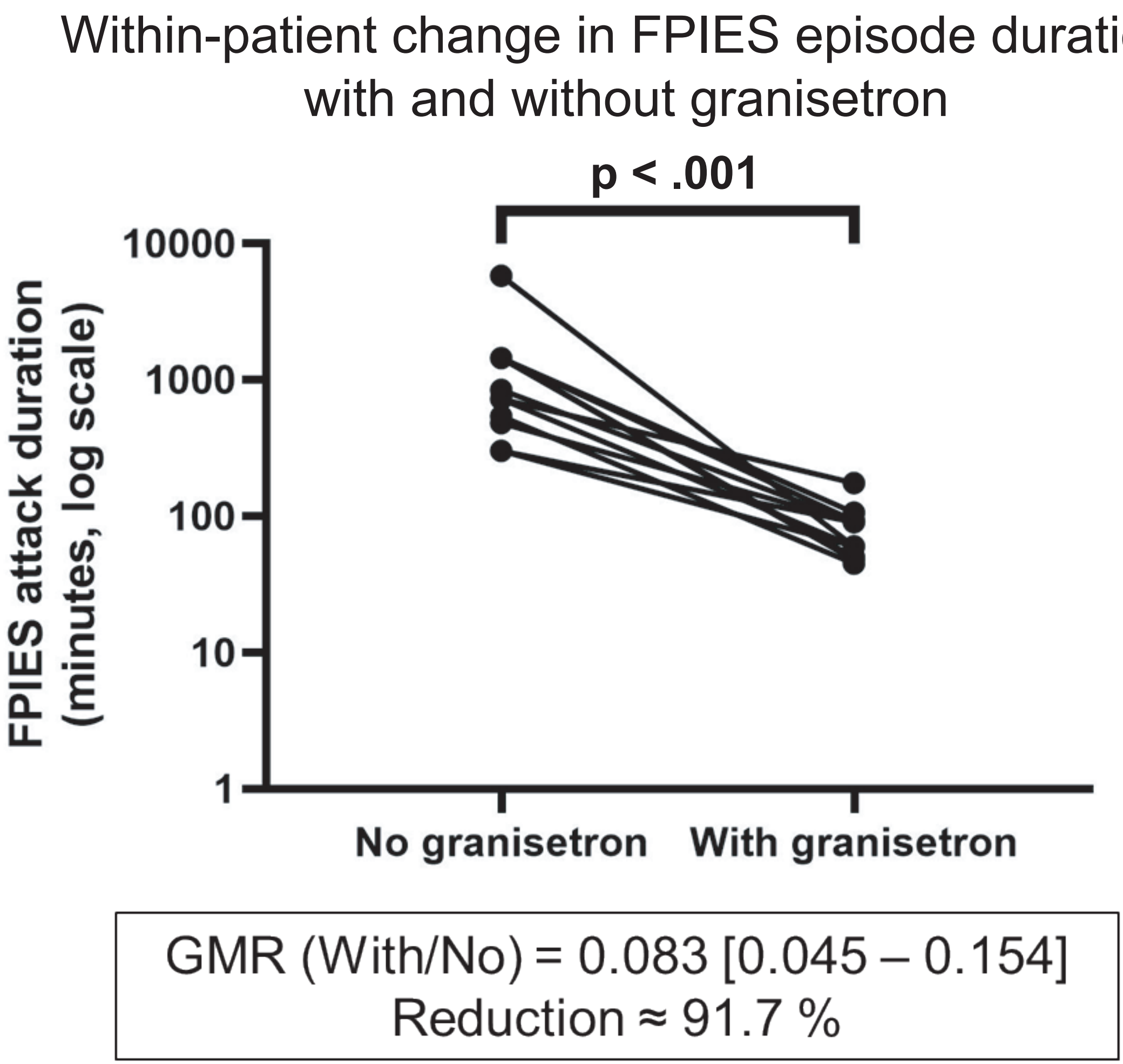

Supplemental Figure 1. Exploratory within-patient comparison of total episode duration with and without granisetron.
Paired comparisons are shown for 12 patients with available data. Episode duration with granisetron includes both the pre-intake and post-intake periods. GMR indicates the geometric mean ratio of episode duration with versus without granisetron.
