# Supplementary material for: Oral 5-HT3 receptor antagonist for at-home management of acute FPIES episodes in adults: A real-world observational study
Source: World Allergy Organ J. 2026 Sep 22;19(10):101473. doi: 10.1016/j.waojou.2026.101473 (PMC13651486; doi:10.1016/j.waojou.2026.101473)
Supplement: Supplementary file 2 [file mmc2.docx]

 **Supplemental Table 1. De-identified episode-level dataset of granisetron-treated acute FPIES events.**

This supplementary table summarizes individual granisetron-treated episodes (n = 33) in 12 adults with FPIES, including patient identifier, trigger food category, presenting symptoms, interval from symptom onset to granisetron intake, time to complete symptom resolution after intake, and reported adverse effects.

Abbreviations: AP, abdominal pain; AD, abdominal distention; D, diarrhea; N, nausea; V, vomiting.

**Supplemental Table S2. Comparison of clinical manifestations during supervised positive oral food challenges (OFCs) and subsequent accidental home FPIES episodes in seven patients.**

Clinical manifestations during supervised positive OFCs were descriptively compared with those observed during subsequent accidental home FPIES episodes in the same seven patients. For patients with multiple accidental home episodes, each symptom was considered present if it occurred in the majority of recorded episodes.
